# MEDIACONNEX: a multicenter randomised trial based on short message service to reduce suicide attempt recurrence in adolescents

**DOI:** 10.1186/s12888-016-0965-8

**Published:** 2016-07-19

**Authors:** Fabienne Ligier, Bernard Kabuth, Francis Guillemin

**Affiliations:** Université de Lorraine, Université Paris Descartes, EA 4360 APEMAC, Nancy – CHU de Nancy, Hôpital d’enfants, Service de Psychiatrie pour enfants et adolescents, rue du Morvan, 54500 Vandoeuvre-lès-Nancy, France; CHU de Nancy, Hôpital d’enfants, Service de Psychiatrie pour enfants et adolescents, Nancy, France; Université de Lorraine, Université Paris Descartes, EA 4360 APEMAC, Nancy - INSERM CIC-EC 1433 – CHU de Nancy, Service d’épidémiologie et évaluation cliniques, Nancy, France

**Keywords:** Adolescent, Prevention of recurrence, Short message service, Suicide attempt

## Abstract

**Background:**

Suicide attempt among adolescents is a public health problem around the world. The risk of recurrence is high: about 30 % of adolescents. New ways to prevent suicide attempt recurrence being developed for adult suicide attempters include maintaining contact with them, and results are encouraging.

**Methods/Design:**

The MEDIACONNEX study will be a simple blinded, parallel-group, multicenter randomised controlled trial. It will compare usual care alone to a program based on usual care plus short message service (SMS) provided to adolescents who attempt suicide and who receive treatment in pediatric and adolescent psychiatry units at hospitals in eastern France. Adolescents will be recruited over an 18-month period. The intervention will be based on the SMS, involving personalized and evolving text messages, sent on days 7 to 14 and months 1, 2, 4 and 6 after the SA. The primary endpoint will be the recurrence of an SA, with an assessment during 12 months. Secondary endpoints will be the evolution of 1) social networks, 2) depression and 3) health-related quality of life, with an assessment at inclusion and at 6 months.

**Discussion:**

This paper describes the design of MEDIACONNEX, which will assess the effectiveness of an SMS program for adolescent suicide attempters on SA recurrence. This program will be easy to reproduce and inexpensive.

**Trial registration:**

The study was registered at ClinicalTrials.gov (no. NCT02762734) on March 2016.

## Background

In industrialised countries, the incidence of suicide attempts (SAs) among adolescents 10–19 years old [1] is estimated to be at 7 to 9 % [2, 3]. Almost one third of these adolescents will make another SA within 1 year. A previous SA may be “an independent and powerful predictor of future attempts”. This “crescendo model of suicidality” concept was developed by Wong [4]: once an SA occurs, the risk of another attempt is increased. Moreover, one of the risk factors of SAs is a first attempt, in particular during the year preceding the attempt. To prevent suicide, we must prevent SAs and particularly their recurrence.

In general, research of adolescent suicide attempters has focused on risk or protective factors [5–7]. However, despite knowledge of these characteristics, the rate of SA recurrence has not decreased. So, care proposals must be creative to limit the recurrence. These last decades, new ways to prevent SA recurrence are being developed for adult suicide attempters. These new ways may be divided into 2 categories: intensive and connectedness care [8]. Intensive care includes specific therapies such as cognitive-behaviour or dialectical behavior therapy [9–12], partial hospitalization [13], and brief psychological interventions at patients’ homes [14]. With some of these treatments, the rate or recurrence has decreased: SA recurrences were reduced 50 % with than without dialectical behavior therapy [11] and the same trend was found in the Weinberg or Fonagy study [12, 13]. Connectedness care involves keeping in touch with patients and encouraging them to call in case of crises in order to avoid the SA recurrence. Motto et al. were pioneers in the field of connectedness care at the end of the 1970s by sending letters to suicide attempters at a regular frequency after the SA [15]. The care may also involve sending postcards [16, 17], making phone calls [18, 19] or using short message service (SMS) [20]. As for intensive care, this care may have positive results on the rate of recurrence or the number of recurrences. In the study of Evans, risk of SA recurrence was reduced for patients who received versus did not receive postcards (odds ratio 0.64). In the Vaiva study, for patients with this care, SA recurrence was reduced 10 % as compared with others in the first 6 months after the SA. These different care modalities have diverse results depending on the sex or personality of the patient [21].

Both types of care have clinical relevance, but the connectedness care is easy to use, has low cost and seems appropriate for adolescents. Indeed, the therapeutic alliance with young patients is not easy to achieve, and intensive care might prevent them from relating to the care. Moreover, in another study in Nancy, France, being lost to follow-up by caregivers was a risk factor of SA recurrence, even 10 years after the SA [22]. Thus, keeping in touch with adolescents after an SA is important, and adolescents may easily accept the idea if presented in a medium they often use: SMS. Some MobilHealth programs have shown good results with the system for adolescents as for adults in other specialties [23, 24].

The MEDIACONNEX study proposes to assess the effectiveness of a new way of connectedness care for adolescents after an SA: it is based on SMS sent over 6 months after the SA to allow adolescents to access care more easily and so limit the risk of SA recurrence.

## Methods/Design

### Aims and hypothesis

We hypothesized that the SMS sent over a 6-month period after an SA, in addition to usual care, may increase the delay in SA reccurence. Therefore, the primary objective is to determine whether, as compared with usual care, SMS in addition to usual care can be used to keep in touch with adolescent suicide attempters to reduce the delay in recurrence of an SA. A secondary hypothesis is that the SMS received after a first SA will reduce the suicide rate.

Secondary objectives are to determine whether, as compared with usual care, SMS in addition to usual care can be used to keep in touch with adolescent suicide attempters to improve the evolution of 1) their social network, 2) depression and 3) health-related quality of life.

### Design

The MEDIACONNEX study will be a simple blinded, parallel-group multicenter randomised controlled trial (RCT) conducted in eastern France. It will compare a program of usual care plus SMS to one of usual care only provided to adolescents who attempted suicide and who receive treatment in a pediatric and adolescent psychiatry unit at a hospital in eastern France (ClinicalTrials.gov no. NCT02762734).

### Setting

Centers of inclusion will be CHU Besançon, CHU Dijon, CHR Metz-Thionville, CHU Nancy, CHU Reims, and CHU Strasbourg. The University Hospital of Nancy will supervise the study and the evaluations will be carried out by the Clinical Investigation Center.

### Participants and consent

All adolescents receiving treatment after an SA in a pediatric or psychiatric unit of participating hospitals will be eligible for inclusion. An SA is defined as “a non-fatal act in which the individual liberally causes self-injury or ingests a substance in excess of any prescribed or generally recognised therapeutic dosage” [25]. Criteria of inclusion are age 13 to 17 years and receiving treatment for an SA. Informed consent will be obtained from patients and/or their parents. Patients will be excluded if they refuse to give consent, cannot participate (somatic or understandability reasons), are incarcerated, or do not have a cell phone.

Adolescents will be recruited during a psychiatric evaluation within 24 h of admission to the pediatric or psychiatric unit of the participating hospitals.

### Randomisation

The Zelen randomisation method will be used. This method, developed in 1979, including consent from patients with evaluated care and for the other group, has been chosen for ethical and legal reasons [26–28]. This method has been preferred to others to limit the differences between the 2 groups due to the risk of intention bias from the physician on the one hand and risk of secondary interruption of participation in the group without SMS on in the other. In each participating hospital, when an adolescent suicide attempter is eligible, a member of the research team will receive a centralized computer-generated random number to allocate the patient to a group. This patient will benefit from usual care plus SMS (group MEDIA) or usual care alone (group CLASSIC). Then, this member of the research team will inform the adolescent's psychiatrist, and the psychiatrist will verify the inclusion criteria and present to the patient and the parents the design of the study for the group to which the patient has been allocated and will gather the informed consent.

### Procedure

The physician will collect data on sociodemographic characteristics, previous SA, psychiatric disease (anxiety, depression etc.) and treatments.

### Intervention

**Fig. 1:**
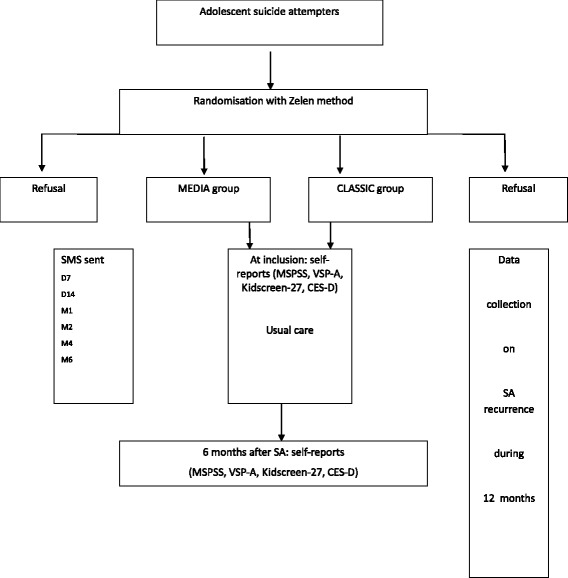
Design of the MEDIACONNEX study. Legends: D = day; M = month; SA = suicide attempt; SMS = short message service

The MEDIA group will receive a “keeping in touch” intervention involving the SMS. The patient will receive 6 SMS items during 6 months after the SA, the first one 7 days after the SA and the last one at 6 months (Fig. 1).

The first SMS will be sent soon after the SA because studies of adult suicide attempters underline the importance of an early start for connectedness measures.

The SMS for this purpose was developed in a focus group of peers and adolescents. A pilot study was carried out in 2015 (MEDIADO, manuscript submitted) to refine the content of the SMS. The SMS item will be sent automatically from a computerized program and will change over the follow-up. The items will be personalized with the surname and genre of the patient and name of the psychiatrist or the immediate superior who meets the patient after the SA. The phone number of the unit will be included in the message and patients will be encouraged to call whenever they need to. The message will specify that someone will be always present for the patient.

Participants of both groups (MEDIA and CLASSIC) will receive usual care, and 4 self-reports will be completed by participants, at inclusion and at 6 months, to evaluate the evolution of the scores for perception of the social network (Multidimensional Scale of Perceived Social Support [MSPSS]), health-related quality of life (Kidscreen-27; Vécu et Santé Perçue des Adolescents [VSP-A]) and mood disorder (Center for Epidemiologic Studies Depression Scale [CES-D]).

The MSPSS is a 12-item self-reporting questionnaire related to the participant’s social network with 5 possible answers for each item. It assesses perceptions of social support from family members, friends, and significant others. It has been used in other countries [29, 30] and was translated and validated in French by a team in Toulouse [31]. The 3 subscales, each addressing a different source of support, were identified and found to have strong factorial validity. The MSPSS had also a good internal and test–retest reliability and moderate construct validity. High levels of perceived social support are associated with low levels of depression and anxiety.

The Kidscreen-27 is a 27-item self-reporting health-related quality-of-life questionnaire developed by a European group [32] and validated in 12 European countries. It evaluates physical wellness, psychological wellness, relationships with parents, the social network, relationships with friends, and schooling. The internal consistency is good and the instrument has good discriminating power.

The VSP-A is a French-language, 37-item self-reporting, health-related quality-of-life questionnaire with 10 dimensions. This instrument has a good internal consistency and good construct validity and content validity [33].

The CES-D is a 20-item self-report on depression, validated in French for adolescents. This instrument has a good internal consistency [34, 35].

### Control intervention

Patients will receive usual care.

### Outcomes

The primary outcome will be the delay of SA recurrence during 12 months after inclusion in the study. The SA recurrence and date of occurrence will be collected by a member of the research team in each participating hospital.

Secondary outcomes will include evaluation and evolution for 6 months after the SA of the adolescent’s social network by using the MSPSS, health-related quality of life by using Kidscreen-27 and VSP-A, and depression by using the CES-D. The scores and their evolution will be compared between CLASSIC and MEDIA groups.

Finally, the entire process will be evaluated by a short self-report at 6 months after the SA.

## Sample size calculation

The sample size of this study was calculated by using data from earlier studies [4, 5]. With one control per experimental subject, an accrual interval of 18 months, and additional follow-up after the accrual interval of 12 months, an alpha risk of 5 % and power of 80 %, assuming 30 % SA recurrence in the first year after an SA and median SA recurrence at 3 months, and an estimated hazard ratio of 0.66, we will need 92 subjects in each group. Assuming 10 % lost to follow-up, we will aim to enroll 112 subjects in each group. This sample size was calculated by using Power Sample Size v3.0.43 for Windows.

## Statistical method

The primary analyses will be conducted on an intent-to-treat basis with data for all randomised participants. A per-protocol analysis of the primary outcome, adjusted on center, will also be reported. Continuous variables will be described with mean (SD) or median (range) and categorical variables with number (%), as appropriate. The chi-square or Fisher exact test will be used for analysis of qualitative variables and Student *t* or Mann–Whitney test for quantitative variables.

Differences in primary and secondary outcomes between the 2 groups will be tested independently. No formal adjustments will be used to constrain the overall type I error associated with the secondary analyses. Secondary analysis aims to supplement evidence from the confirmatory primary analysis, and results will be interpreted in this context.

Data will by analyzed by using SAS 9.3 for Windows (SAS Inst., Cary, NC). *P* < 0.05 will be considered statistically significant.

### Primary outcome analysis

To identify differences in SA recurrence, a time-to-event analysis with Cox regression modeling will be used. The study period will be up to 12 months after the SA of inclusion. Patients without another SA, who die, or who are lost to follow-up will be censored. Patients with and without an SA during the study period will be compared between the MEDIA and CLASSIC groups. Factors associated with SA recurrence will be compared by log rank test, with *p* < 0.05 considered statistically significant. Cox proportional hazards modelling of SA recurrence will include sex, group and other variables with *p* < 0.2 on univariate analysis (i.e., explanatory variables). The score obtained from the CES-D at the time of inclusion will be considered an explanatory variable. This method will allow for considering a confusion factor and determining which initial characteristics are significant risk factors for recurrence of SA. Hazard ratios (HRs) and 95 % confidence intervals (95 % CIs) will be calculated to indicate the probability of SA recurrence by characteristics entered in the model. We will test for interactions between the included variables. Each variable will be checked for proportionality assumption by a graphical method and the Moreau method, and variables not meeting proportionality will be used for stratification [36].

### Secondary outcomes analysis

The weight of the explanatory variables on SA recurrence will be compared between the MEDIA and CLASSIC groups; the log-rank test will be used for comparing the time of SA recurrence between the groups for each explanatory variable. The groups will be compared for scores obtained with the MSPSS, Kidscreen-27 and VSP-A questionnaires.

## Ethics and permissions

The protocol is in accordance with the Declaration of Helsinki and has been approved by French Ministry of Health. Informed consent will be obtained from patients and/or their parents. The study is ongoing in terms of planning and organization and inclusions will begin in a few weeks.

## Discussion

This randomised trial has been planned to assess the effect of a program of “connectedness care” for adolescent suicide attempters on SA recurrence, which will represent an advance in care for this young population. This program will be easy to develop in France and other countries, for reasonable cost.

## Abbreviations

SA, suicide attempt; SMS, short text message
